# V64M Mutation in Leukemia Inhibitory Factor Gene in Women Infertility

**Published:** 2017-07

**Authors:** Gholamreza NIAEI, Bita AMIR TAGHAVI, Amir NIAEI

**Affiliations:** 1. Dept. of Biology, Faculty of Sciences, University of Guilan, Rasht, Iran; 2. Dept. of Genetics, Islamic Azad University, Tehran Medical Branch, Tehran, Iran; 3. Dept. of Internal Medicine, School of Medicine, Tehran University of Medical Sciences, Tehran, Iran

## Dear Editor-in-Chief

Infertility is one of the major increasing gynecological problems worldwide. The incidence of infertility varies between populations mainly due their dietary, lifestyle, environmental and occupational factors, and infectious diseases. Embryonic implantation takes place on the uterine inner surface after a series of molecular and cellular interactions between the embryo and endometrium.

Various different cytokines are suggested just before participating in successful implantation, including uterine natural killer cells, leukemia inhibitory factor (LIF) and interleukin 15 (IL-15) (1–3). Leukemia inhibitory factor (LIF), a pleiotropic cytokine from interleukin-(IL-) 6 families, regulates various cellular functions via binding to membrane-bound LIF receptor (LIFR) and gp130. The role of LIF in human implantation is less clear. LIF probably plays a role in endometrial function in humans and domestic species. Progesterone is a major regulator of LIF expression and coincides with progesterone dominance (3–5). The LIF gene mutation, G to A transition at position 3400 in exon 3 leading to valine to methionine exchange at codon 64 (V64M), in the AB loop region of the LIF protein (5–7). The prevalence of LIF gene mutations was investigated in a population of infertile women and found a significantly higher frequency of the functionally relevant mutation (7). The role of the LIF gene allele and the hormonal stimulation were hypothesized, the aim of the study was to determine whether a diagnosed cause of infertility is directly related to 3400 G>A LIF gene mutation, reflected in women with various causes of infertility.

From April 2015 to October 2015, we screened 60 non-selected infertile women and 80 fertile controls with at least one live birth for LIF gene mutations in Tabriz University of Medical Science, Tabriz, Iran. Genomic DNA was extracted from human peripheral blood cells by the “salting out” technique (8). Then, single strand conformation polymorphism (SSCP) and direct sequence analysis of these aberrantly migrating bands were carried out for investigation of gene LIF V64M mutation.

In infertile women LIF gene mutations which are statistically significantly (*P*<0.05) connected to women with infertility and more frequent than in healthy women (Table 1). The 3400 G>A LIF gene Mutation may be associated with increased risk of idiopathic women infertility. Therefore, our study indicates that there is significant association between the LIF gene V64M Mutation and biological activity of LIF and cause implantation failure.

**Table 1:** Prevalence of the 3400 G>A LIF gene mutation in patient and control groups and evaluation of their association with infertility

**Polymorphism**	**Normal**		**Heterozygote**		**Homozygote**		***P* value**
	**Case (%)**	**Control (%)**	**Case (%)**	**Control (%)**	**Case (%)**	**Control (%)**	
LIF 3400 G/A	8.1	13.8	59.9	75.5	32	10.7	0.006

This study was limited to study of the role of only one single cytokine, LIF. It is the cumulative effect of different cytokines acting on the embryo and the endometrium, essential for successful implantation.
